# 2195. Impact of Optimizing Urine Culture Susceptibility Reports on Prescribing of Antimicrobials in Outpatients at the Atlanta VA Health Care System (AVAHCS)

**DOI:** 10.1093/ofid/ofad500.1817

**Published:** 2023-11-27

**Authors:** Christopher Gale, Tiffany Goolsby, Andrew S Webster

**Affiliations:** Department of Veterans Affairs, Atlanta, Georgia; Atlanta VAMC, Atlanta, Georgia; Atlanta VA Medical Center, Decatur, Georgia

## Abstract

**Background:**

To encourage appropriate prescribing in uncomplicated urinary tract infections (UTIs), the AVAHCS optimized urine susceptibility reports by adding comments guiding selection of narrow-spectrum beta-lactams for Enterococcus spp and certain Enterobacterales (E. coli, K. pneumoniae, Proteus spp) and hiding the susceptibility of levofloxacin for Enterococcus spp. The purpose of this study is to determine the impact of these changes on antibiotic selection in outpatients.

**Methods:**

This study was a retrospective chart review of patients with positive urine cultures for the target organisms from March - December 2019 and March - December 2022. Patients were included if they had urine cultures that resulted with one of the target organisms and received UTI treatment. Those excluded had positive blood cultures or were hospitalized at the time of sample collection, had a previous urine culture or received treatment for UTI within 30 days, had a urological procedure in the preceding 12 months, or had a polymicrobial culture result. The primary outcome was the percent change in the use of quinolones before and after implementation. Secondary outcomes included the percent change in the use of aminopenicillins and cephalosporins for Enterococcus spp and Enterobacterales, respectively.

**Results:**

For Enterococcus spp, there was a 29.2% decrease in the selection of quinolones as definitive therapy. There was a 37.1% increase in the use of aminopenicillins as definitive therapy. For Enterobacterales, there was a 15% decrease in the selection of quinolones as definitive therapy. There was a 13.3% increase in the use of first-generation cephalosporins as definitive therapy.

**Conclusion:**

For Enterococcus spp UTI, following suppression of levofloxacin from the C&S reports and addition of provider comments, quinolones were less likely to be prescribed and aminopenicillins were more likely to be prescribed. In Enterobacterales UTI, following addition of prescriber comments, quinolones were less likely to be prescribed and first-generation cephalosporins were more likely to be prescribed, though the increase was less pronounced than was seen in the Enterococcus spp group. These results suggest the combination of susceptibility suppression and comments was more impactful than a comment alone.

**Disclosures:**

**All Authors**: No reported disclosures

